# CRISPR/Cas9: implication for modeling and therapy of amyotrophic lateral sclerosis

**DOI:** 10.3389/fnins.2023.1223777

**Published:** 2023-07-06

**Authors:** Yajun Shi, Yan Zhao, Likui Lu, Qinqin Gao, Dongyi Yu, Miao Sun

**Affiliations:** ^1^Key Laboratory of Birth Defect Prevention and Genetic Medicine of Shandong Health Commission, Key Laboratory of Birth Regulation and Control Technology of National Health Commission of China, Center for Medical Genetics and Prenatal Diagnosis, Shandong Provincial Maternal and Child Health Care Hospital Affiliated to Qingdao University, Jinan, Shandong, China; ^2^Institute for Fetology, the First Affiliated Hospital of Soochow University, Suzhou, Jiangsu, China

**Keywords:** amyotrophic lateral sclerosis, gene editing, CRISPR/Cas9, ALS models, gene therapeutic, clinical trials

## Abstract

Amyotrophic lateral sclerosis (ALS) is a deadly neurological disease with a complicated and variable pathophysiology yet to be fully understood. There is currently no effective treatment available to either slow or terminate it. However, recent advances in ALS genomics have linked genes to phenotypes, encouraging the creation of novel therapeutic approaches and giving researchers more tools to create efficient animal models. Genetically engineered rodent models replicating ALS disease pathology have a high predictive value for translational research. This review addresses the history of the evolution of gene editing tools, the most recent ALS disease models, and the application of CRISPR/Cas9 against ALS disease.

## Introduction

Amyotrophic lateral sclerosis (ALS), also known as Lou Gehrig’s disease, is a progressive neurodegenerative disease that destroys motor neurons (MNs) in the spinal cord, brainstem, and motor cortex. ALS within 2–5 years of the onset of symptoms, leading to muscle weakening, atrophy, paralysis, respiratory failure, and death (Marcus, 2022). Additionally, ALS is one of the most common adult motor neuron diseases, affecting 2–3 persons out of every 100,000 individuals globally (Oskarsson et al., 2018; Feldman et al., 2022). Evidence shows that the prevalence of ALS has been rising in recent years, which may be largely attributed to the advancements in clinical services that allow precise diagnosis (Logroscino et al., 2018). However, ALS is still incurable. Current medications, such as Riluzole, prolong patient survival by an average of 3 months but are only modestly beneficial, highlighting the need for solutions to treat this dangerous condition.

Most cases of ALS are sporadic and have an unknown etiology, whereas approximately 10 ~ 15% of patients have a family history of the disease, strongly indicating a genetic predisposition (Sahdeo and Goldstein, 2021). The genetic etiology of ALS has been linked to more than 50 genes that include causal or disease-modifying variations (Taylor et al., 2016; Giovannelli et al., 2023). The most commonly investigated ALS genes, including superoxide dismutase (*SOD1*), chromosome 9 open reading frame 72 (*C9orf72*), TAR DNA-binding protein 43 (*TDP43*), and RNA binding protein fused in sarcoma (*FUS*), account for roughly 75% of familial ALS cases (Owens, 2017; Fang et al., 2022). Our understanding of the pathophysiology of ALS has significantly increased due to the analysis of the molecular pathways underlying these mutant ALS genes, revealing new information about prospective therapeutic targets. Accordingly, developing models of ALS and targeting the mutant genes (i.e., gene therapy) has been investigated worldwide to translate these gene therapies into the clinical setting.

With a corrected pathogenetic mutation, gene editing offers a potential treatment for ALS, and it is now beginning to be used in clinical settings (Mullard, 2019). In particular, the genome-editing technology known as clustered regularly interspaced short palindromic repeats/CRISPR-associated system 9 (CRISPR/Cas9) has gained attention for its possible benefits in the management and treatment of ALS (Cho et al., 2013; Yamashita et al., 2013). Compared to other genome editing techniques like transcription activator-like effector nucleases (TALENs) and zinc finger nucleases (ZFNs), CRISPR/Cas9 is easy to use and reasonably priced (Yang et al., 2017; Bhardwaj et al., 2022; Meijboom et al., 2022). In this review, we emphasize the history of the emergence of gene editing tools and highlight the developments in CRISPR/Cas9 mediated gene correction and disease models gene therapy for ALS disease.

## The evolution and history of gene editing

Gene editing constantly evolves to provide more precise, diverse, and large-scale implementations, allowing us to gain innovative insights into the biological field. Previously, homologous recombination (HR) and non-homologous end-joining (NHEJ) were used to carry out genetic engineering. Due to its low efficacy in many cell types and animals, we still need another alternative. Currently, there exist three main gene editing technologies: ZNFs, TALENs, and the CRISPR/Cas system, among which CRISPR/Cas system is now the most predominant technology.

### Zinc finger nucleases

*Flavobacterium okeanokoites* (*FokI*) and zinc finger structure, which promoted further development of gene editing, were discovered in the 1990s (Li et al., 1992). Zinc finger proteins region with conserved sequences are arranged in a certain order, containing three to six Cys2-His2 fingers, followed by attachment of *FokI* to the end of the protein. Zinc fingers are transcription factors, where each finger recognizes 3–4 bases. Ultimately, ZFNs recognized a 9–18 bp sequence (Liu et al., 1997). After ZFNs bind to DNA in DNA-binding domains, the *FokI* nuclease induces cleavage as a dimer, resulting in double-stranded breaks (DSB) in the target loci. Then, HR and NHEJ are activated to complete gene editing through the intracellular DNA repair mechanism (Nakashima et al., 2022). Theoretically, ZFNs recognize almost all 64 possible nucleotide triplets, but the specificity and affinity of ZNFs are still issues. Additionally, ZFNs are reported to exert a significant cytotoxic effect because of the off-target cleavages (Gupta et al., 2013).

### Transcription activator-like effector nucleases

Like ZFNs, TALENs are artificial chimeric proteins engineered by fusing a non-specific *FokI* endonucleases domain to a DNA-binding domain recognizing an arbitrary base sequence (Zhang et al., 2019). The TALENs are well known for their unique DNA-binding domain in their central region, conserved tandem between 7 and 34 homologously repeated amino acid residues. Two residues located at positions 12 and 13, called repeat variable di-residues (RVD), are highly variable residues responsible for recognizing a specific nucleotide (Agarwal and Gupta, 2021).

Unlike ZFNs, TALENs had the advantage that they could bind to just one nucleotide inside its DNA-binding domains rather than three, resulting in enhanced specificity and decreasing in the off-target events (Table 1). TALENs, cheaper, safer, more efficient, and easier to engineer than zinc-finger proteins, can be applied more widely to life sciences than ZFNs (Huo et al., 2019). This gene-editing tool was utilized for various cell lines, including induced pluripotent stem cells (iPSCs) (Kwon et al., 2021; Qiu et al., 2021; Siles et al., 2023), nematodes (Verdu et al., 2020, 2021), zebrafish (Zhu et al., 2021; Sertori et al., 2022), and generating knockouts in rodent models (Lee et al., 2021). Despite the improvement, simplification, and high specificity of TALENs, they have an obvious limitation: being quite large (impeding delivery). This limitation makes it difficult to deliver and express a pair of TALENs into the cells of interest because they cannot be packaged in vectors of their limited cargo capacity, such as adeno-associated viruses (AAVs), or delivered as RNA molecules (Khan, 2019; Jo et al., 2022).

**Table 1:** Comparison of the three currently used gene editing platforms: ZFN, TALEN, and CRISPR/Cas9.

	ZFN	TALEN	CRISPR/Cas9
Structure	Dimer	Dimer	Monomer
Modification pattern	Fok1 nuclease	Fok1 nuclease	Cas 9 nuclease
DNA binding molecule	Zinc finger protein	Transcription activator-like effectors	sgRNA or crRNA
Target sequence size	Typically 9–18 bp per ZFN monomer, 18–36 bp per ZFN pair	Typically 14–20 bp per TALEN monomer, 28–40 bp per ZFN pair	22 bp: Typically 20 bp guide sequence +2 bp PAM sequence
Targeting limitations	Difficult to target non-G-rich site	5′ targeted base must be a T for each TALEN monomer	The targeted site must precede a PAM sequence
Specificity	Tolerating a small number of positional mismatches	Tolerating a small number of positional mismatches	Tolerating multiple consecutive/positional mismatches
DNA recognition mechanism	Protein-DNA interactions that introduce DSB	Protein-DNA interactions that introduce DSB	RNA-guided protein-DNA interactions that introduce DSB
Multiplex genome editing	Not easy (few models)	Not easy (few models)	Easy (high-yield multiplexing available)
Delivery vehicle	Easy via electroporation and viral vectors transduction	Easy *in vitro* delivery; difficult *in vivo* due to the large size of TALEN DNA and their high probability of recombination	Easy *in vitro*; the moderate difficulty of delivery *in vivo* due to poor packaging of the large Cas9 by viral vectors
Off-target effects	Highly possible off-target activities	Low possible off-target activities	Variable; limited off-target activities
Cytotoxicity	Variable to high	Low	Low
Cost	Low	High	Low
Popularity	Low	Moderate	High

### CRISPR/Cas system

**Figure 1:**
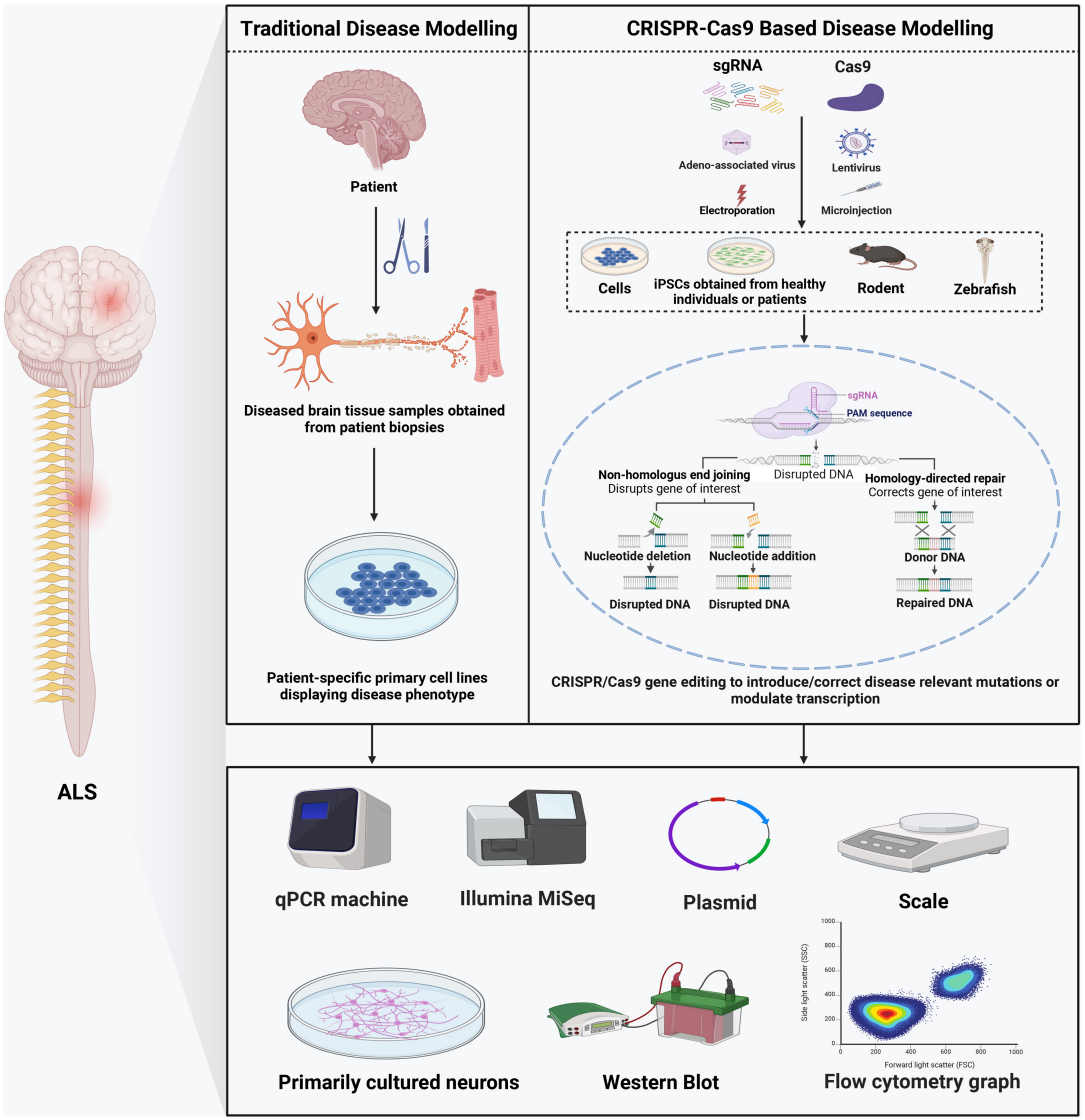
Comparison of traditional and CRISPR/Cas9-based disease modeling.

The CRISPR/Cas system, whose gene editing ability was confirmed in human cells in 2013 (Cho et al., 2013), is the most recent platform in genome editing. Unlike nucleases that recognize the target sequence through protein-DNA interaction, CRISPR-Cas systems target sequences through RNA and DNA base pairing (Zhang et al., 2019) (Table 1). Naturally occurring CRISPR-Cas systems can be categorized into two classes based on the *Cas* genes’ structural variations and their organization styles (Makarova et al., 2015). The type II CRISPR/Cas9 system, which depends on a single Cas protein from *streptococcus pyogenes* (SpCas9) targeting particular DNA sequences and has been adopted for gene editing applications, is the most frequently used subtype of CRISPR systems (Jiang et al., 2013). Mechanistically, CRISPR/Cas9 systems have two components: single-guide RNA (sgRNA) and Cas9 endonuclease. Usually, the sgRNA was designed near the adjacent protospacer motif (PAM) and bound to the target DNA sequence by Watson–Crick base pairing. Then Cas9 precisely cleaves the DNA to generate a DSB (Hu et al., 2018). These DSBs are commonly repaired by NHEJ or HDR, resulting in gene disruption and inactivation of the targeted gene (Lee et al., 2016) (Figure 1). The sgRNA and Cas protein-coding gene are usually delivered via transfection and packaging into AAVs or lentivirus (Kenjo et al., 2021; Ling et al., 2021; Nahmad et al., 2022).

Although there are still several issues that remain to be resolved in CRISPR/Cas9 system, including well-known off-target effects, cytotoxicity with DNA damage, chromosomal rearrangement and frequent p53 activation (Hendriks et al., 2020; Bravo et al., 2022). However, compared to other genome editing technologies, CRISPR/Cas9 system is easier, more scalable, and more efficient (Li et al., 2020). Therefore, it is frequently used to create animal models or to treat serious, incurable human diseases (Li et al., 2021; Baskoylu et al., 2022).

Although promising, this strategy raises potential safety concerns as it relies on repairing the DSBs and NHEJ created by CRISPR/Cas9, which may cause deleterious large deletions, chromosomal rearrangements, or undesirable mutagenic outcomes (Zhao et al., 2022). To overcome this limitation and improve the safety and applicability of the CRISPR/Cas9 system, a new paradigm-shifting class of genome editing technology, CRISPR single-base editors, have recently been developed. CRISPR single-base editors may precisely and directly insert point mutations into chromosomal DNA without creating DSBs by combining the Cas9 nickase with nucleobase deaminases (Caso and Davies, 2022). To date, two main kinds of base editors have been developed: adenine base editors (ABEs), which catalyze A/T to G/C conversions, and cytosine base editors (CBEs), which catalyze the conversion of G/C base pairs to T/A base pairs (Anzalone et al., 2020). However, base editors cannot make multiple base pair insertions and deletions. When many As or Cs are present around the target site, they might inadvertently become mutated and created off-target effects. To overcome this limitation, termed prime editing, which could insert longer stretches of DNA, has been developed recently. Base editors have been applied to various cell types and organisms, including animal models of human genetic diseases (Kawamata et al., 2023).

## Amyotrophic lateral sclerosis model construction

Given that there are no ethical considerations, the experimental time is relatively short, and the expense is minimal, cell models are commonly used to explore many neurological illnesses, including ALS. Furthermore, recent advances in CRISPR/Cas9 technology allow researchers to precisely target and introduce modifications in the genomic DNA of different cell lines (Kramer et al., 2018; Karginov et al., 2023). The introduction of the CRISPR/Cas9 system has facilitated the development of numerous ALS cell models, such as HT22, iPSCs, BV2, Neuro 2a, NSC-34, hESC, and HeLa cell lines, etc. For example, Liu et al. (2022) recently used CRISPR/Cas9 gene-editing technology to create an astrocyte elevated gene-1 (AEG-1) deficient HT22 cell model that can be used to study AEG-1 related ALS. In addition, Zhang et al. (2022) used CRISPR/Cas9 to introduce a UBQLN2 P497H mutation into a healthy control iPSC line. Following this, the mutated iPSC line could differentiate into motor neurons, which can help comprehend the pathology of UBQLN2-related ALS (Zhang et al., 2022). Importantly, CRISPR-iPSC-based disease modeling could expedite research on ALS (Sen and Thummer, 2022) by targeting *SOD1* (Kim et al., 2020), *TDP-43* (Burkhardt et al., 2013), *FUS* (Wang et al., 2017), and *C9ORF72* (Cali et al., 2019; Ababneh et al., 2020; Krishnan et al., 2020) genes, etc. Furthermore, Strohm et al. (2022) also made CRISPR/Cas9-engineered HeLa cells with ALS UBQLN2 mutations to determine how UBQLN2 mutations cause ALS. ALS can be caused by OPTN gene mutations, which result in cytoplasmic mislocalization, ubiquitination, and accumulation of nuclear TDP-43. Prtenjaca et al. (2022) used CRISPR/Cas9 methodology to create OPTN knockout (KO) cell lines for Neuro 2a and NSC-34 neurons and BV2 microglia to investigate the relationship between optineurin and TDP-43. Their research showed that OPTN mutations might lead to elevated levels of TDP-43 protein in microglia when optineurin is absent (Prtenjaca et al., 2022). In addition, Zhang et al. (2022) created a heterozygous *FUS*-Q290X knock in the human embryonic stem cell (hESC) line, which will be a valuable tool for investigating the disease mechanisms of ALS using the CRISPR/Cas9 system.

CRISPR/Cas9 technology is not only helpful in creating ALS cell models but also in creating ALS animal models. For instance, ALS can be caused by mutations in valosin-containing protein (VCP). On the other hand, there is no constitutive VCP knockout animal model due to embryonic lethality. Voisard et al. (2022) developed a constitutive CRISPR/Cas9-induced VCP knockout zebrafish model. Furthermore, Wall et al. (2021) created the Drosophila knockin models using the CRISPR/Cas9 technique to investigate VCP diseases (including multisystem proteinopathy and ALS) (Wall et al., 2021). To investigate the pathogenic effects of TP73 mutations in ALS, Russell et al. (2021) utilized CRISPR/Cas9 technology targeting TP73 in zebrafish and assessed motor neuron quantity and axonal structure. Their findings revealed that knocking out TP73 in zebrafish using CRISPR/Cas9 resulted in disrupted motor neuron development and abnormal axonal morphology, indicative of ALS pathology (Russell et al., 2021). Additionally, Bose et al. (2019) developed a TDP-43 loss-of-function model in zebrafish using the CRISPR/Cas9 system. Moreover, to examine mechanisms underlying mutations in *FUS* that lead to neuronal dysfunction in ALS patients, the *C. elegans* knockin models were generated by Baskoylu et al. (2022) using CRISPR-Cas9-mediated genome editing. Their findings suggested impaired autophagy might play a role in the neuromuscular defects and protein homeostasis associated with ALS *FUS* knockin animals (Baskoylu et al., 2022). As for the FUS gene, Zhang et al. (2018) developed a novel knockin rat model expressing a *Fus* point mutation (R521C) via CRISPR/Cas9. They discovered that FUS might regulate sleep, circadian rhythms, and learning and memory behavior (Zhang et al., 2018). Degeneration of neurons in many ALS subtypes has been linked to DNA damage and defective repair, but the underlying mechanisms are unclear. The role of *TDP-43* in DNA damage response (DDR) has not been explored, despite growing evidence suggesting the involvement of other RNA/DNA binding proteins like *FUS* in DDR (Wang et al., 2018). Mitra et al. (2019) demonstrated that the loss of nuclear *TDP-43* is linked to DNA double-strand break repair defects and DDR signaling in ALS by using CRISPR/Cas9-mediated conditional depletion of TDP-43 in *C. elegans*. Also, Baskoylu et al. (2018) made ALS knockin models in *C. elegans* by editing the endogenous *Sod-1* gene with CRISPR/Cas9 and discovered that knockin models could replicate the neurotransmitter-type specificity of ALS. Using the CRISPR/Cas9 system, Kao et al. (2020) created MATR3 S85C knockin mice that mimic early-stage ALS and would be useful in future basic and preclinical research. Sporadic ALS patients were found to have mutations in the *CREST* gene (also called SS18L1), which acts as a calcium-regulated transcriptional activator. Loss of *CREST* causes neuroinflammatory responses and ALS-like motor defects in mice, as demonstrated by the work of Cheng et al. (2019), who used the CRISPR/Cas9 system to create *CREST* knockout and Q394X knockin mice. In addition, *C9orf72*-deficient mice (Sullivan et al., 2016) have been successfully established by using the CRISPR/Cas9 system due to the recent discovery of hexanucleotide repeat expansion in the C9orf72 gene as a major cause of frontotemporal lobar degeneration (FTLD) with ALS (DeJesus-Hernandez et al., 2011).

Researching the etiology and developing treatments for human diseases requires animal models. The discovery of specific gene mutations that cause neurodegenerative disorders has led to the development of numerous models in small animals with symptoms similar to those experienced by people with the diseases. Genetically modified rodents are today’s most widely used animal models because they apply to explore pathogenesis. However, most genetically modified rodent models do not exhibit overt neurodegeneration, creating difficulties in using them to rigorously test the effects of therapies on neurodegeneration (Philips and Rothstein, 2015). Recent research using CRISPR/Cas9-targeted large animals (such as pigs and monkeys) (Li et al., 2021; Yang et al., 2022) has uncovered important pathological events that resemble neurodegeneration in the patient’s brain. Still, these events could not be produced in small animal models. Injections of multiple sgRNAs are more effective than injections of a single type of sgRNA at a high concentration for achieving complete gene knockout. The complete gene knockout by the CRISPR/Cas9 system is conducive to reducing the test times of large animals and ensuring their accuracy, which is strongly supported by ethical considerations (Zuo et al., 2017). Although no ALS models in large animals have been developed so far using CRISPR/Cas9 technology, it is essential to create a large animal ALS model using this approach to gain more knowledge about the pathogenesis of ALS.

## The application of the CRISPR/Cas9 technology for amyotrophic lateral sclerosis therapy

CRISPR/Cas9 system, used in cells and animal models, is a promising tool for modeling and treating genetic diseases (Table 2). Compared with cellular-level studies, animal models are more favorable for exploring the pathogenic gene and molecular pathways of diseases at the organismal level. For ALS, definite treatments are still lacking. Here, we mainly focus on CRISPR/Cas9 system applications in ALS. The great advantage of this strategy is that correction of the mutant DNA eliminates abnormal downstream pathways and is a one-time intervention theoretically. Currently, limited studies have been accomplished in the ALS field.

**Table 2:** Studies of the applications of CRISPR/Cas9-mediated genome editing in ALS.

Target	Point mutation	Organism/cell line	Delivery	Results	References
*SOD1*	A272C	Human ALS patient-derived iPSCs	Electroporation	Discovered early biomarkers and pathways dysregulated in ALS	(Wang et al., 2017)
	L144FVX	Human ALS patient-derived iPSCs	Adenoassociated Virus Vector	Over half of the iPSCs targeted the Src/c-Abl signaling pathway, suggesting that Src/c-Abl may be a potentially useful target for developing new drugs to treat ALS	(Imamura et al., 2017)
	E100G	Human ALS patient-derived iPSCs	Nucleofection	Identified activated ERK and JNK signaling are critical drivers of neurodegeneration in mutant *SOD1* motor neurons	(Bhinge et al., 2017)
	G93A	*SOD1*-G93A transgenic mouse	Adenoassociated Virus Vector	Disruption of mutant *SOD1* enhances the survival of spinal cord motor neurons and improves motor function and life span	(Gaj et al., 2017)
	G93A	*SOD1*-G93A transgenic mouse	Use an AAV vector and inject it into the lumbar subarachnoid space	Deleting mutant *SOD1* via CRISPR/Cas9 prolongs survival in an ALS mouse model	(Duan et al., 2020)
	G93A	*SOD1*-G93A transgenic mouse	Intrathecal injection	The mouse treated by split-intein CRISPR base editor had a reduced rate of muscle atrophy, improved neuromuscular function, and up to 40% fewer SOD1 immunoreactive inclusions	(Lim et al., 2020)
	G93A	Human *SOD1*-G93A missense mutation iPSCs	Sendai virus-based vector	The mutant motor neurons accumulated misfolded and aggregated forms of *SOD1* in cell bodies, causing axonopathy and aberrant neurotransmission	(Kim et al., 2020)
	G93A	*SOD1*-G93A transgenic mouse	Intracerebroventricular injection	Rescues motor function deficits and extends survival in a *SOD1*-ALS mouse model	(Chen et al., 2022)
*C9ORF72*	Deleted GGGGCC	Human ALS patient-derived iPSCs	Cloned into px300 plasmid and introduced to the iPSCs by nucleofection	Provides a proof-of-principle for using CRISPR-Cas9-mediated excision of the pathogenic *C9orf72* repeat expansion as a therapeutic strategy in ALS	(Lopez-Gonzalez et al., 2016)
	Deleted GGGGCC	Human ALS patient-derived iPSCs	Nucleofection	The mutations lead to increased Ca^2+^-permeable and enhance selective MNs’ vulnerability to excitotoxicity	(Selvaraj et al., 2018)
	Deleted GGGGCC	*C9ORF72* patient-derived iPSCs	Sendai virus-based vector	Performed an extensive phenotypic characterization of ALS-iPSCs-derived MNs	(Dafinca et al., 2016)
	Deleted GGGGCC	*C9ORF72* patient-derived iPSCs	Nucleofection	Proved partial inhibition of an overactivated DNA repair pathway suppresses a cell death pathway is the pathology of ALS	(Lopez-Gonzalez et al., 2019)
	Deleted GGGGCC	*C9ORF72* patient-derived iPSCs	Adenoassociated Virus Vector 9	The CRISPR/Cas9-mediated genome correction reduced RNA foci, poly-dipeptides, and haploinsufficiency, major hallmarks of ALS	(Meijboom et al., 2022)
*FUS*	R521H	*FUS* patient-derived iPSCs	Nucleofection	Mutations in *FUS* result in apparent defects in MNs derived from *FUS*-ALS patients	(Guo et al., 2017)
	G1566A	*FUS* patient-derived iPSCs	Electroporation	Discovered early biomarkers and pathways dysregulated in ALS	(Wang et al., 2017)
	H517Q	Human ALS patient-derived iPSCs	Nucleofection	Identified activated ERK and JNK signaling as critical drivers of neurodegeneration in mutant FUS MNs	(Bhinge et al., 2017)
	R521H, P525L	Human ALS patient-derived iPSCs	Nucleofection	Uncovered a pathway of defective DNA ligation in *FUS*-linked ALS	(Wang et al., 2018)
	R521H, P525L	Human ALS patient-derived iPSCs	Nucleofection	Metabolic dysfunction is not the underlying cause of the ALS-related phenotypes previously observed in these MNs	(Vandoorne et al., 2019)
	R524S, P525L	*C. legan* knockin models	Nucleofection	Autophagy dysfunction likely contributes to protein homeostasis and neuromuscular defects in ALS *FUS* knockin animals	(Baskoylu et al., 2022)
*TARDBP*	M337V	*TARDBP-*M337V patient-derived iPSCs	Nucleofection	The abnormal function of BDNF may explain the aberrant TDP-43 activity	(Tann et al., 2019)
	M337V	*TARDBP-*M337V patient-derived iPSCs	Sendai virus-based vector	The MNs with *TARDBP* mutations impaired mitochondrial Ca^2+^ uptake contributes to glutamate excitotoxicity	(Dafinca et al., 2020)

ALS, amyotrophic lateral sclerosis; MNs, motor neurons; iPSCs, induced pluripotent stem cells.

### SOD1

Several cells and animal models targeted on *SOD1* have been reported. A sgRNA was applied to disrupt *SOD1* via the facial vein, leading to decreased expression of *SOD1* protein in the spinal cord, reduced muscle atrophy, and delayed disease onset in mice models. With the improvement of motor neuron function, the survival time increased by 28–30 days (Gaj et al., 2017). Other favorable results of *SOD1* decrease in the spinal cord were reported in three studies, which showed increased survivability (Lim et al., 2020; Chen et al., 2022). The CRISPR/Cas9 technology that can be used to validate the efficacy of rescuing iPSC-derived motor neuron cells from the harmful effects of the *SOD1* mutation has been described in several articles (Kim et al., 2020; Deng et al., 2021). Wang et al. (2017) generated two human iPSC lines from ALS patients, *SOD1^+/A272C^* and *SOD1^+/C14T^* heterozygous disease-causing mutations. Then, they generated their respective isogenic disease-free iPSCs by CRISPR/Cas9 mediated gene correction (Wang et al., 2017). This study may help identify biomarkers and pathways for ALS and offers new evidence for ALS therapy. Additionally, a recent study showed that CRISPR/Cas9-mediated gene editing is an effective strategy to target *SOD1*, leading to a disease-free condition in two ALS mouse models (Deng et al., 2021). The main limitation of these studies is that the mice were treated at a young age before exhibiting symptoms of ALS. Therefore, the treatment’s effectiveness would be uncertain in older mice with ALS phenotype.

In drug screening assay, Imamura et al. (2017) used CRISPR/Cas9 mediated *SOD1* corrected iPSCs lines generated from one ALS patient carrying the L144FVX mutation in the *SOD1* gene and homologous arms corrected mutation as a control. The corrected cell line demonstrated a reversal of *SOD1*-related pathologic phenotypes, including increased motor neuron survival, reserved internal ATP level, and breakdown of misfolded SOD1 protein. They also found that bosutinib, one of the specific Src/c-Abl inhibitors, could modestly extend the survival of the SOD1 mutation mouse model of ALS, suggesting that Src/c-Abl may be a potential target for developing new drugs to treat ALS (Imamura et al., 2017).

### C9ORF72

*C9orf72* mutation, leading to an expansion of GGGGCC (G4C2) hexanucleotide repeat expansion (HRE) in the first intron, is the most significant gene discovery for ALS (Gijselinck et al., 2012). The size of the hexanucleotide sequence is less than 24 repeats in a healthy person but can number in the thousands of repetitions in affected individuals (Iacoangeli et al., 2019). Multiple studies have demonstrated that the *C9orf72* mutations affect subsequent protein synthesis and cause premature neuronal death by causing harmful either a gain or loss of function, such as impaired clearance of dipeptide proteins and excitotoxicity from the accumulation of glutamate receptors (Ghasemi et al., 2021; Meijboom et al., 2022). Therefore, reducing the expression of *C9orf72* is a potentially promising target in halting ALS disease.

The field of gene editing, especially the CRISPR/Cas9 system, offers potential treatment modalities for ALS pathology by physically excising the repeat expansion mutation from *C9orf72* at the genetic level. Meijboom et al. (2022) administer AAV9 vectors containing gRNA and Cas9 into the bilateral striatum of aged 2–3 months *C9orf72*-associated mice, which rescues partial ALS phenotypes. Respectively, two separate studies also reported the utility of CRISPR/Cas9 in targeting either (G4C2) repeat DNA (Pinto et al., 2017) or RNA to reduce repeat RNA transcription and decreased myotonia in the ALS mouse model. These findings are consistent with other reports demonstrating the positive benefit of excising the HRE in *C9orf72* (Ababneh et al., 2020; Piao et al., 2022), indicating potentially viable strategies for therapeutic intervention.

### FUS

The mutation of the *FUS* gene, a DNA/RNA-binding protein, in chromosome 16 was discovered as a causative factor for ALS in 2009. Serval of mutations in the *FUS* gene have been found in motor neurons of ALS patients, like C1574T, A1564G, G1566A, etc. (Lattante et al., 2013; Du et al., 2015), and these mutations cause up to 4% of familial ALS (Mackenzie et al., 2010). Investigating the gene editing of *FUS* by the CRISPR/Cas9 system will provide a platform for revealing novel therapeutic entry points.

Wang et al. (2017) first successfully corrected the *FUS^+/G1566A^* mutations in ALS iPSCs by CRISPR/Cas9 system, and the repaired ALS iPSCs possessed normal pluripotency. Furthermore, Bhinge et al. (2017) obtained iPSCs from ALS patients for recessive H517Q mutation in the *FUS* gene and corrected this recessive mutation to wild-type using the CRISPR/Cas9 system. Their results confirmed that both p38 and ERK kinase are activated in mutant *FUS*, indicating that MAPK activation is a key pathway in *FUS* mutation in ALS (Bhinge et al., 2017). Additionally, Guo et al. (2017) proved that the different mutations in the *FUS* gene of motor neurons caused typical cytoplasmic hyperexcitability and progressive axonal transport defects, which were rescued by CRISPR/Cas9-mediated genetic correction of the *FUS* mutation in patient-derived iPSCs. As the above results indicated, CRISPR/Cas9 system provides a platform for revealing novel therapeutic entry points.

### TARDBP

Although the specific mechanisms by which TDP-43 protein, encoded by *TARDBP*, cause ALS is unknown, numerous studies have shown that a substantial fraction of ALS patients features abnormalities in TDP-43, whose function losses in the nucleus and gain of toxic in the cytoplasm (Kuo et al., 2014; De Conti et al., 2015). In addition, TDP-43 may involve stress granule formation and regulation of neurite growth (Shelkovnikova et al., 2018; Zuo et al., 2021; McMillan et al., 2023), and the reduction of cytoplasmic TDP-43 levels can rescue neurons from death (Walker et al., 2015). Brown et al. (2022) revealed evidence that TDP-43 is a promising therapeutic target for ALS.

Currently, the application of CRISPR/Cas9-mediated genetic correction targeting *TARDBP* gene therapy is limited. To investigate the mechanisms of *TARDBP* mutations in ALS, Tann et al. (2019) confirmed that hippocampal CA1-specific absence of TDP-43 enhanced the expression of *Sod1* mRNA and reduced dendrite complexity and spine. They also used CRISPR/Cas9 to correct M337V in three iPSC lines from ALS patients, and then these iPSCs were differentiated into excitatory cortical/hippocampal-like neurons. Their study indicated that M337V mutation in *TARDB*P is a direct cause of impaired BDNF secretion, essential for neuron survival, differentiation, and synaptic plasticity (Tann et al., 2019).

## Clinical trials on ALS worldwide

Although, four therapies (riluzole, edaravone, sodium phenylbutyrate and taurursodiol, and dextromethorphan-quinidine) have been approved now, but they could not stops or reverses disease progression (Meijboom and Brown, 2022). Using ALS as the search term, 969 clinical trials were gathered from clini-caltrials.gov on 20 June 2023. Unfortunately, the bulk of ALS clinical trials have failed to show any promising outcomes during the past two decades. This is partially caused by the therapeutic agents’ low bioavailability, ineffective therapeutic delivery to the CNS, difficulties with therapeutic administration, dearth of clinically relevant disease models, lack of late diagnosis, effective biomarkers, and inadequate understanding of the underlying molecular mechanisms causing the disease (Johnson et al., 2022). Therefore, new therapies and diagnostics are urgently needed to help with therapy and early diagnosis. All the current worldwide Phase III ALS clinical trials that are being summarized in Table 3.

**Table 3:** Ongoing Phase III Clinical Trials on ALS Worldwide.

Clinical title	Study title	Agents	Locations
NCT04297683	Healey ALS platform trial-master protocol	Zilucoplan, Verdiperstat	United States
NCT05568615	Extension study following the studies MT-1186-A03 or A04 to evaluate the safety of oral edaravone in subjects with ALS	Edaravone MT-1186	Japan
NCT05151471	Efficacy and safety extension study of oral edaravone administered in subjects with ALS	Edaravone MT-1186	United States
NCT04768972	A study to evaluate the efficacy, safety, pharmacokinetics and pharmacodynamics of ION363 in amyotrophic lateral sclerosis participants with fused in sarcoma mutations	ION363	United States
NCT05193994	Triumeq in amyotrophic lateral sclerosis	Dolutegravir, Abacavir, Lamivudine	Australia
NCT04745299	Evaluation the efficacy and safety of mutiple lenzumestrocel treatment in patients with ALS	Riluzole, Lenzumestrocel	Korea
NCT05866926	A multicenter, open-label extension study to investigate the long-term safety of FAB122 in patients with ALS	FAB122	Barcelona, Spain
NCT03127267	Efficacy and safety of masitinib vs. placebo in the treatment of ALS patients	Masitinib, Riluzole	United States
NCT05753852	Open Label extension of TUDCA-ALS study	Tauroursodeoxycholic	France
NCT04856982	A study of BIIB067 (Tofersen) initiated in clinically presymptomatic adults with a confirmed superoxide dismutase 1 mutation	Tofersen	United States
NCT04220190	RAPA-501 therapy for ALS	RAPA-501 autologous T cells	United States
NCT04057898	Evaluation of MN-166 (Ibudilast) for 12 months followed by an open-label extension for 6 months in patients with ALS	MN-166	United States
NCT04302870	Motor neurone disease-systematic multi-arm adaptive randomized trial	Trazodone, Memantine, Amantadine	United States
NCT05156320	Efficacy and safety of apitegromab in patients with later-onset spinal muscular atrophy treated with nusinersen or risdiplam	Apitegromab	United States

## Conclusions and future

We discussed numerous recent studies that have implicated gene editing, especially CRISPR/Cas9, to investigate the pathophysiology of ALS by animal models or iPSCs in this review. There remain limitations with these approaches, such as off-target effects, limited size of delivery vehicle, and low efficiency of gene correction through the HDR mechanism (Modell et al., 2022).

Over 50 genes, in which *SOD1*, *C9orf72*, *TARDBP*, and *FUS* are commonly genetic alterations, are listed as ALS-associated genes. Studies using CRISPR/Cas9-mediated *in vivo* genome editing of *SOD1*, *C9orf72*, *TARDBP*, and *FUS* functions show that this technique can identify the underlying molecular mechanism and reverse the pathologic phenotype in patient-derived disease models. These studies suggest that CRISPR/Cas9 system may play a potential role in gene therapy for ALS. However, more thorough studies are still needed to investigate the various underlying mechanisms of ALS to determine the treatment for ALS.

## Author contributions

YS, YZ, and LL wrote the manuscript with support from QG, DY, and MS. All authors contributed to the article and approved the submitted version.

## Funding

This work was supported by grants from the National Key R&D Program of China (2022YFC2703700 and 2019YFA0802600) and the National Natural Science Foundation of China (81974244 and 81570960).

## Conflict of interest

The authors declare that the research was conducted in the absence of any commercial or financial relationships that could be construed as a potential conflict of interest.

## Publisher’s note

All claims expressed in this article are solely those of the authors and do not necessarily represent those of their affiliated organizations, or those of the publisher, the editors and the reviewers. Any product that may be evaluated in this article, or claim that may be made by its manufacturer, is not guaranteed or endorsed by the publisher.
